# Pharmacological and non-pharmacological interventions in patients undergoing nasal surgeries for prevention of emergence agitation: a systematic review and network meta-analysis

**DOI:** 10.1016/j.bjane.2024.844565

**Published:** 2024-10-16

**Authors:** Gustavo R.M. Wegner, Bruno F.M. Wegner, Henrik G. Oliveira, Luis A. Costa, Luigi W. Spagnol, Valentine W. Spagnol, Gilberto T.F. de Oliveira Filho

**Affiliations:** a Universidade Federal da Fronteira Sul (UFFS), Faculdade de Medicina, Passo Fundo, RS, Brazil; b Universidade Federal do Rio Grande do Sul (UFRGS), Faculdade de Medicina, Porto Alegre, RS, Brazil

**Keywords:** General anesthesia, Anesthesia recovery period, Nasal surgical procedures, Emergence agitation

## Abstract

**Background:**

Emergence agitation is a common complication after nasal surgeries, marked by increased agitation and a heightened risk of injuries. Factors like urinary catheter, endotracheal tube, postoperative pain, and younger age contribute to its occurrence. Due to the variety of preventive approaches reported in the literature, a network meta-analysis is essential.

**Methods:**

This systematic review employs a network meta-analysis design, following Cochrane Handbook and PRISMA-NMA criteria. Inclusion criteria involve randomized controlled studies on pharmacological and non-pharmacological interventions for preventing emergence agitation in nasal surgeries. Electronic searches, including PubMed, Scopus, Embase, Cochrane Library, and Web of Science, without language or date restrictions, were conducted. Two independent reviewers selected studies, and data extraction was performed using standardized tables. Bayesian NMA, MetaInsight web app, and Cochrane Foundation Risk of Bias Assessment Tool were applied for data analysis and bias assessment.

**Results:**

After a rigorous selection process, 17 Randomized Controlled Trials (RCTs) encompassing 2,122 patients and 14 interventions were included. The best ranked treatments identified were intraoperative dexmedetomidine (1 μg.kg-^1^ for 10 minutes as a bolus, followed by 0.4 μg.kg^-1^.h^-1^), bilateral nasociliary and maxillary nerve block, ketamine (0.5 mg.kg^-1^ administered 20 minutes before the end of surgery), nasal compression for 40 minutes before anesthesia induction, and suction above the cuff of the endotracheal tube.

**Conclusions:**

Both pharmacological and non-pharmacological interventions emerged as effective strategies in mitigating emergence agitation after nasal surgeries, offering clinicians valuable options for improving postoperative outcomes in this patient population.

## Introduction

Postanesthetic emergence agitation, also called emergence delirium, is a common complication after nasal surgeries, leading to increased risks of injury, pain, bleeding, and self-extubation. The most commonly known risk factors are the presence of urinary catheters, endotracheal tubes, postoperative pain, younger age, and inhalation anesthesia. With prevalence rates ranging from 22.2% to 55.4% in nasal surgeries, emergence agitation poses significant challenges to patient safety and adds substantial costs to healthcare.^1^ The costs arise from prolonged hospital stays and the need for additional interventions, highlighting the importance of identifying effective preventive measures.

Understanding the physiopathology of emergence agitation is crucial for developing targeted interventions. The literature suggests a multifactorial interaction, with disruption of the cholinergic system being a central component.^2^ Other contributing factors include neurotransmitter imbalances, inflammatory dysregulations, and stress responses.^3^

Preventive strategies for emergence agitation have been preliminarily explored. Dexmedetomidine shows promise due to its ability to modulate the inflammatory response and mitigate hyperactivity of the sympathetic nervous system, essential for preventing acute postoperative cognitive changes.^4^^,^^5^ Other pharmacological agents, such as ketamine and butorphanol, have also been investigated for their potential to reduce the incidence of emergence agitation.^6^, ^7^, ^8^

In addition to pharmacological approaches, regional anesthetic blocks may help prevent emergence agitation by minimizing the need for systemic agents.^9^, ^10^, ^11^ Furthermore, non-pharmacological interventions have been associated with lower incidences of emergence agitation, representing cost-effective therapeutic tools that could improve clinical outcomes.^12^, ^13^, ^14^

The diverse approaches proposed in the literature for preventing emergence agitation necessitate a comprehensive, systematic analysis. Network meta-analysis is a valuable tool for this purpose, as it allows for direct and indirect comparisons of intervention effectiveness and establishes a relative hierarchy.^15^ Consequently, our analysis aims to differentiate between the various explored interventions and their efficacy in preventing emergence agitation, potentially providing a solid foundation for enhancing perioperative management and clinical outcomes for patients undergoing nasal surgeries.

## Methods

This systematic review and network meta-analysis were conducted and reported based on the Cochrane Handbook and to the PRISMA Extension Statement for Reporting of Systematic Reviews Incorporating Network Meta-analyses of Health Care Interventions.^16^^,^^17^ The review protocol was registered on PROSPERO (CRD42023464926).

### Eligibility criteria

Inclusion in this meta-analysis was restricted to studies that met all the following eligibility criteria: (1) Randomized controlled studies; (2) Those describing pharmacological or non-pharmacological interventions for prevention of emergence agitation; (3) Those in patients American Society of Anesthesiologists (ASA) physical status I or II undergoing nasal surgeries; and (4) Those with emergence agitation assessed by the Riker Sedation/Agitation Scale or the Richmond Agitation Sedation Scale. We excluded studies: (1) With insufficient information or data that could not be retrieved by contacting the corresponding authors; (2) That did not present interventions suitable for comparison in the network meta-analysis; and (3) That did not meet the transitivity assumption.

### Search strategy

We conducted a comprehensive literature search across multiple electronic databases, including MEDLINE, Scopus, Embase, Cochrane Library, and Web of Science. The search was conducted on June 11^th^, 2024. There was no restriction on language or publication date.

The search strategy consisted of: ("Nasal surgery" OR "Nose surgery" OR "Nasal endoscopy" OR "Nasoendoscopy" OR "endoscopic sinus" OR "Sinus surgery" OR "Rhinoplasty" OR "Sinuplasty" OR "Septoplasty" OR "Turbinoplasty" OR "Nasal polyp removal" OR "Nasal polypectomy" OR "Nasal polyp resection" OR "Ethmoidectomy" OR "Rhinoseptoplasty") AND ("Delirium" OR "Emergence delirium" OR "Emergence agitation" OR "Postoperative delirium" OR "Post-operative delirium" OR "Post operative delirium" OR "Postoperative agitation" OR "Post-operative agitation" OR "Post operative agitation" OR "Postanesthesia delirium" OR "Post-anesthesia delirium" OR "Post anesthesia delirium" OR "Postanaesthesia delirium" OR "Post-anaesthesia delirium" OR "Post anaesthesia delirium" OR "Postanesthesia agitation" OR "Post-anesthesia agitation" OR "Post anesthesia agitation" OR "Postanaesthesia agitation" OR "Post-anaesthesia agitation" OR "Post anaesthesia agitation").

The included documents were exported to a reference manager (Mendeley 1.19.8®) to remove duplicates. The reference lists of all the included articles were also reviewed for potential citation eligibility.

### Selection of studies

Two reviewers (GRMW and HGO) independently performed the two-step selection. Titles and abstracts of all results were initially screened; after this, the full text of the selected studies was obtained and independently assessed for eligibility. In cases of disagreement, a third reviewer was consulted.

### Data extraction and synthesis

Data extraction was performed in duplicate using standardized data extraction tables in Google Sheets containing article identification, sample numbers, characteristics, and outcome measures. The data was initially collected in an inclusive way, and the relevant data was then synthesized by generating new tables for better comprehension. After the preliminary search, a network meta-analysis approach was considered appropriate.

### Risk of bias assessment

Quality assessment of all the Randomized Controlled Trials (RCTs) was performed with Cochrane's tool, wherein studies are scored as high, low, or unclear risk of bias in 5 domains: selection, performance, detection, attrition, and reporting.^18^

### Data analysis

A Network Meta-Analysis (NMA) was conducted utilizing a Bayesian NMA model with the assumption of evidence consistency. We estimated and reported risk ratios along with their associated 95% Bayesian credible intervals. Treatments were ranked using rank probabilities, and the Surface Under the Cumulative Ranking (SUCRA) and Litmus Rank-O-Gram were calculated in relation to individual outcomes.^19^

The quantitative synthesis was conducted by GRMW using the MetaInsight web app, which incorporated R packages such as “netmeta”, “gemtc”, “BUGSnet”, “rjags”, and “coda”.^20^, ^21^, ^22^, ^23^, ^24^, ^25^ The different interventions covered by the network meta-analysis were mapped onto graphs. Generalized Linear Models (GLMs) with a random-effects model were utilized, based on a priori noninformative distributions, binomial likelihood distributions, and a log link function. Markov Chain Monte Carlo (MCMC) simulations, involving 4 chains, were employed with a burn-in phase of 5,000 iterations followed by 20,000 iterations and a thinning factor of one. Model selection was based on trial and error, guided by MCMC convergence diagnostics, including Gelman-Rubin trace plots and potential scale reduction factors, as well as leverage plots.^26^ Model fit was assessed using the posterior mean of the residual Deviance (Dbar), effective degrees of freedom (pD), and Deviance Information Criterion (DIC), and was visually represented through residual deviance plots of the network Meta-Analysis Model (NMA), Unrelated Mean Effect Model (UME), stem plots, and leverage plots. The precision of estimates was indicated by 95% Credibility Intervals (95% CrIs). A comprehensive explanation of the statistical terms is provided in the supplementary material (Supplementary Methods).

## Results

**Figure 1:**
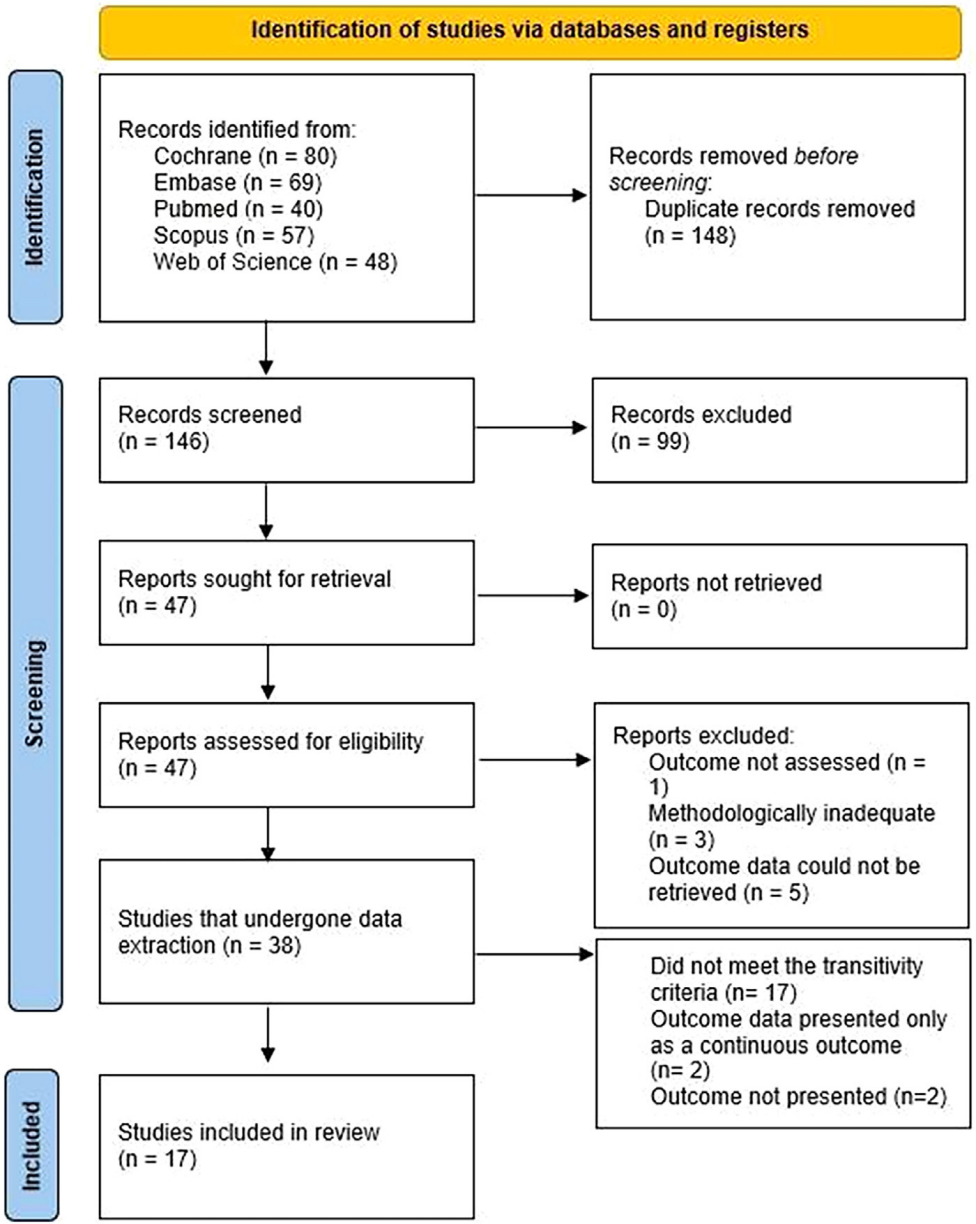
Study Flow Diagram.

The selection process included 294 manuscripts, as presented by the PRISMA flowchart (Fig. 1). After all selection steps, 17 RCTs were included, totaling 2,122 patients and 14 different interventions (Supplementary Table S1). The timing of each intervention administration was better described in the Supplementary Material. Different control settings, such as saline solution administration, placebo, and not performing the intervention, were considered equivalent.

### Network meta-analysis

We conducted a network meta-analysis on patients undergoing nasal surgeries under general anesthesia maintained with inhalational anesthetics. The literature indicates that sevoflurane is a risk factor for emergence agitation in nasal surgeries.^1^^,^^27^ Additionally, a recent meta-analysis identified inhalational anesthesia, particularly sevoflurane, as a risk factor for emergence agitation in adult patients.^28^ Although only inhalational anesthesia with sevoflurane was explicitly identified as a risk factor, the reviewed studies predominantly utilized sevoflurane. For instance, in the cited meta-analysis, out of 12 RCTs comparing TIVA to inhalational anesthetics, 9 studies used sevoflurane. Consequently, the absence of other inhalational agents as identified risk factors may result from limited available evidence. Therefore, we conducted an analysis including articles that utilized inhalation anesthetics for maintenance anesthesia without discriminating between the use of sevoflurane and assessed our findings through two subgroup analyses: one for general anesthesia maintained with sevoflurane and another for general anesthesia maintained with non-sevoflurane inhalational agents.

Regarding other pertinent analyses, intervention analysis on patients undergoing general anesthesia with propofol could not be performed due to the limited and insufficient number of studies. Additionally, a network meta-analysis comparing different anesthetic regimens was not feasible due to the limited number of studies addressing this comparison.

The full details, including the number of patients, number of comparisons, number of studies, and summary statistics of each outcome for each comparison, were reported in supplementary tables. The results for the analyses were summarized in graphs, forest plots, SUCRA plots and Litmus Rank-O-Gram in relation to individual outcomes.

### Inhalation agents for anesthesia maintenance analysis

Seventeen RCTs^6^, ^7^, ^8^, ^9^, ^10^, ^11^, ^12^, ^13^^,^^29^, ^30^, ^31^, ^32^, ^33^, ^34^, ^35^, ^36^ involving 2,122 patients across 14 different interventions were included in the analysis. Of the 91 possible pairwise comparisons, 14 were direct. All studies provided event data, and the network graph was connected. A random-effects model was used for the analysis. Detailed information on the quantitative synthesis is available in Supplementary Tables 1, 2, 3, 4, and Supplementary Figures 1, 2, 3, 4, and 13. The inhalation anesthetic agents studied were Sevoflurane,^7^, ^8^, ^9^, ^10^, ^11^, ^12^^,^^33^^,^^36^ Desflurane,^6^^,^^13^^,^^30^, ^31^, ^32^^,^^34^ and Isoflurane.^14^^,^^29^^,^^35^

### Subgroup analysis

#### Sevoflurane maintenance group

**Figure 2:**
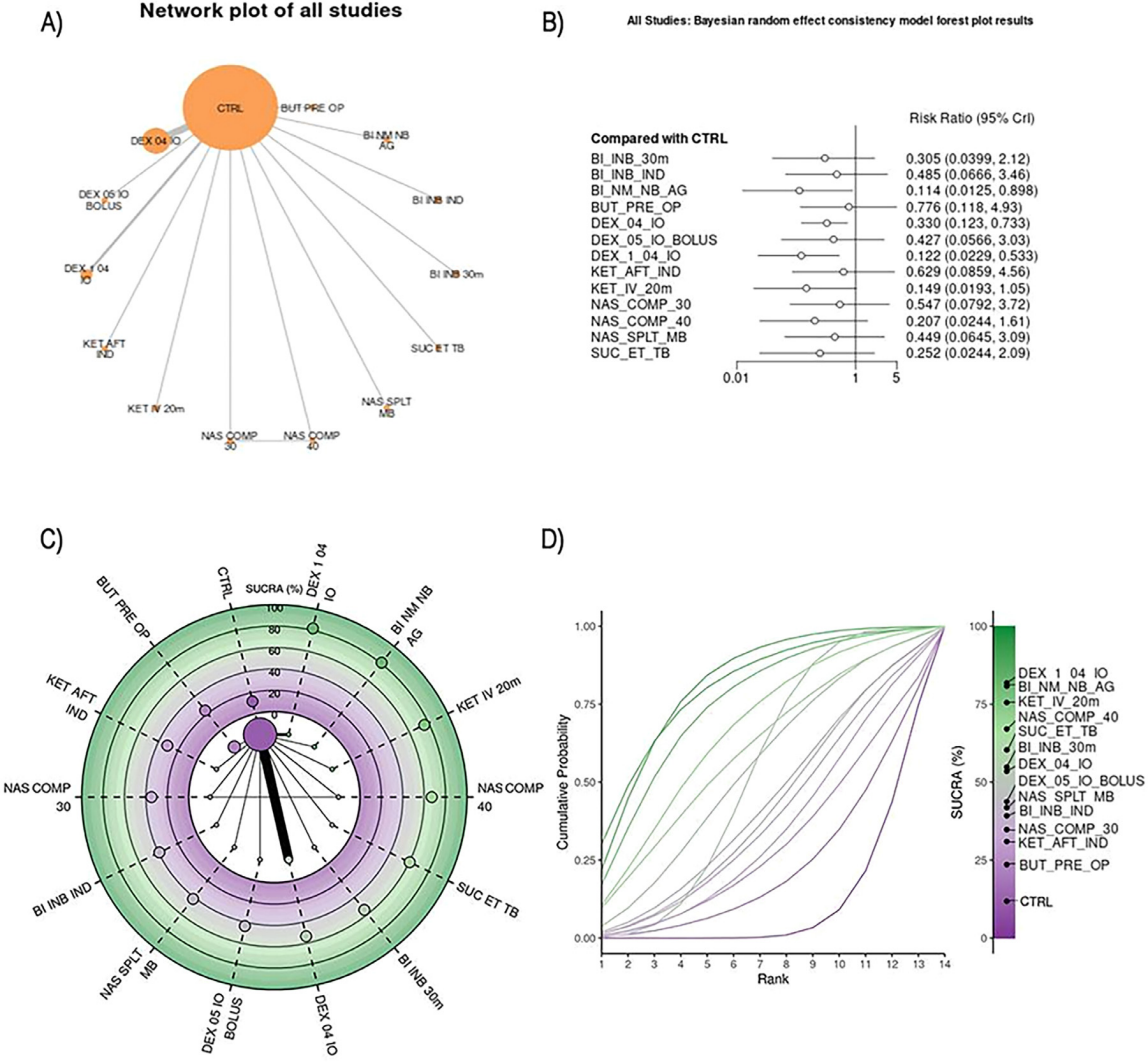
(A) A network plot of the studies included in the analysis that used inhalation agents for anesthesia maintenance. The number of studies that examined a treatment and compared two given treatments is represented by the size of the nodes and the thickness of the edges, respectively. (B) Bayesian random effect consistency model forest plot results. Forest plot for pairwise comparisons of included interventions versus placebo of the studies included in the analysis that used inhalation agents for anesthesia maintenance. The effect size is expressed as risk ratio. (C) Radial Surface Under the Cumulative Ranking Curve (SUCRA) plot of the studies included in the analysis that used inhalation agents for anesthesia maintenance. The treatments are ordered clockwise starting at “12 o'clock,” with the SUCRA value of each treatment plotted radially. The more outward the representation of the intervention, the higher its relative efficacy. The radial SUCRA contains a network of interventions with node sizes proportional to the evidence supporting them. (D) Litmus Rank-O-Gram in relation to individual outcomes of the studies included in the analysis that used inhalation agents for anesthesia maintenance. It shows the cumulative probability for each guidance technique to be ranked as first, second, third and beyond alongside with the SUCRA values. The higher the bar, the greater the probability of ranking in that position. CTRL, Control (Saline, placebo, or procedure not performed); BI_INB_30m, Bilateral Infraorbital and Infratrochlear Nerve Block 30 min before surgery; BI_INB_IND, Bilateral Infraorbital and Infratrochlear Nerve Blocks before induction; BI_NM_NB_AG, Bilateral nasociliary and maxillary nerve block after general anesthesia; BUT_PRE_OP, Preoperative administration of butorphanol 20 mcg.kg^-1^; DEX_04_IO, Intraoperative Dexmedetomidine infusion of 0.4 mcg.kg^-1^.h^-1^; DEX_05_IO_BOLUS, Intraoperative Dexmedetomidine bolus of 0.5 mcg.kg^-1^; DEX_1_0.4_IO, Intraoperative Dexmedetomidine bolus of 1 mcg.kg^-1^ for 10 min followed by infusion of 0.4 mcg.kg^-1^.h^-1^; KET_AFT_IND, Ketamine bolus of 1 mg.kg^-1^ after induction; KET_IV_20m, Ketamine bolus of 0.5 mg kg^-1^ IV 20 min before end of the surgery; NAS_COMP_30, Nasal compression for 30 min before induction of anesthesia; NAS_COMP_40, Nasal compression for 40 min before induction of anesthesia; NAS_SPLT_MB, Nasal splinting and mouth breathing training before procedure; SUC_ET_TB, Suction above Cuff Endotracheal Tube.

Eight RCTs involving 1,275 patients used sevoflurane^7^, ^8^, ^9^, ^10^, ^11^, ^12^^,^^33^^,^^36^ across nine different interventions (Fig. 2). Of the 36 possible pairwise comparisons, eight were direct. All studies had event data, and the network graph was connected. The analysis followed a random-effects model. More details on the quantitative synthesis can be found in Supplementary Tables 1, 2, 5, 6, and Figures 5, 6, 7, 8, and 14.

#### Non-sevoflurane inhalation agents’ maintenance group

**Figure 3:**
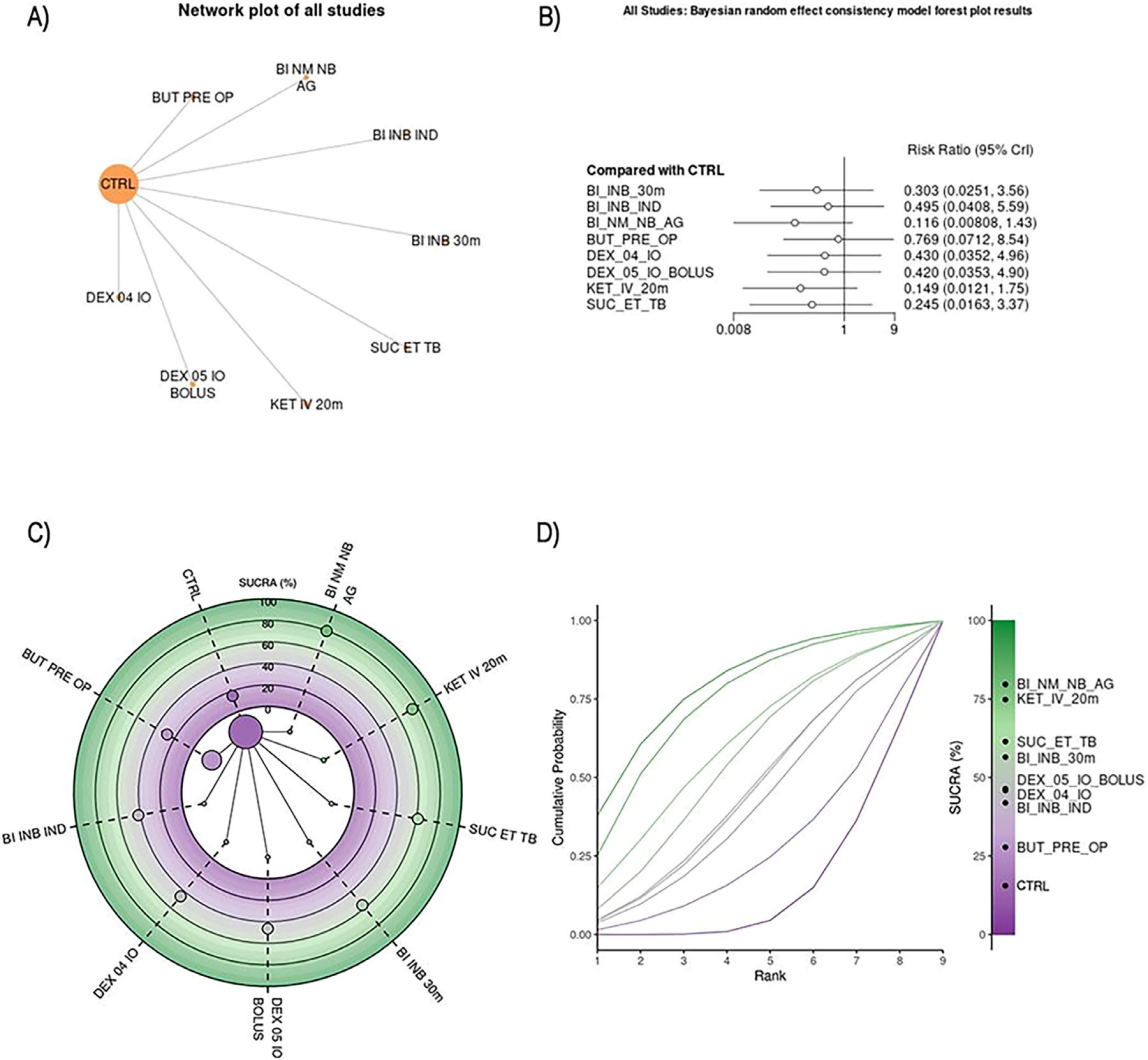
(A) A network plot of the studies included in the analysis that used non-sevoflurane inhalation agents for anesthesia maintenance. The number of studies that examined a treatment and compared two given treatments is represented by the size of the nodes and the thickness of the edges, respectively. (B) Bayesian random effect consistency model forest plot results. Forest plot for pairwise comparisons of included interventions versus placebo of the studies included in the analysis that used non-sevoflurane inhalation agents for anesthesia maintenance. The effect size is expressed as risk ratio. (C) Radial Surface Under the Cumulative Ranking Curve (SUCRA) plot of the studies included in the analysis that used non-sevoflurane inhalation agents for anesthesia maintenance. The treatments are ordered clockwise starting at “12 o'clock,” with the SUCRA value of each treatment plotted radially. The more outward the representation of the intervention, the higher its relative efficacy. The radial SUCRA contains a network of interventions with node sizes proportional to the evidence supporting them. (D) Litmus Rank-O-Gram in relation to individual outcomes of the studies included in the analysis that used non-sevoflurane inhalation agents for anesthesia maintenance. It shows the cumulative probability for each guidance technique to be ranked as first, second, third and beyond alongside with the SUCRA values. The higher the bar, the greater the probability of ranking in that position. CTRL, Control (Saline, placebo, or procedure not performed); KET_IV_20m, ketamine bolus of 0.5 mg.kg^-1^ IV 20 min before end of the surgery; DEX_04_IO, Intraoperative Dexmedetomidine Infusion of 0.4 mcg.kg^-1^.h^-1^; BI_INB_IND, Bilateral Infraorbital and Infratrochlear Nerve Blocks before induction; BI_NM_NB_AG, Bilateral nasociliary and maxillary nerve block after general anesthesia; DEX_05_IO_BOLUS, Intraoperative Dexmedetomidine bolus of 0.5 mcg.kg^-1^; SUC_ET_TB, Suction above Cuff Endotracheal Tube; BUT_PRE_OP, Preoperative administration of butorphanol 20 mcg.kg^-1^.

Nine RCTs involving 864 patients used non-sevoflurane inhalation anesthetics across nine different procedures (Fig. 3).^6^^,^^13^^,^^14^^,^^29^, ^30^, ^31^, ^32^^,^^34^^,^^35^ Of the 21 possible pairwise comparisons, seven were direct. All studies had event data, and the network graph was connected. A random-effects model was used for the analysis. Further information on the quantitative synthesis is available in Supplementary Tables 1, 2, 5, and 6, and Supplementary Figures 9, 10, 11,12, and 15. The inhalation anesthetic agents included were Desflurane,^6^^,^^13^^,^^30^, ^31^, ^32^^,^^34^ and Isoflurane.^14^^,^^29^^,^^35^

### Best ranked treatments

**Figure 4:**
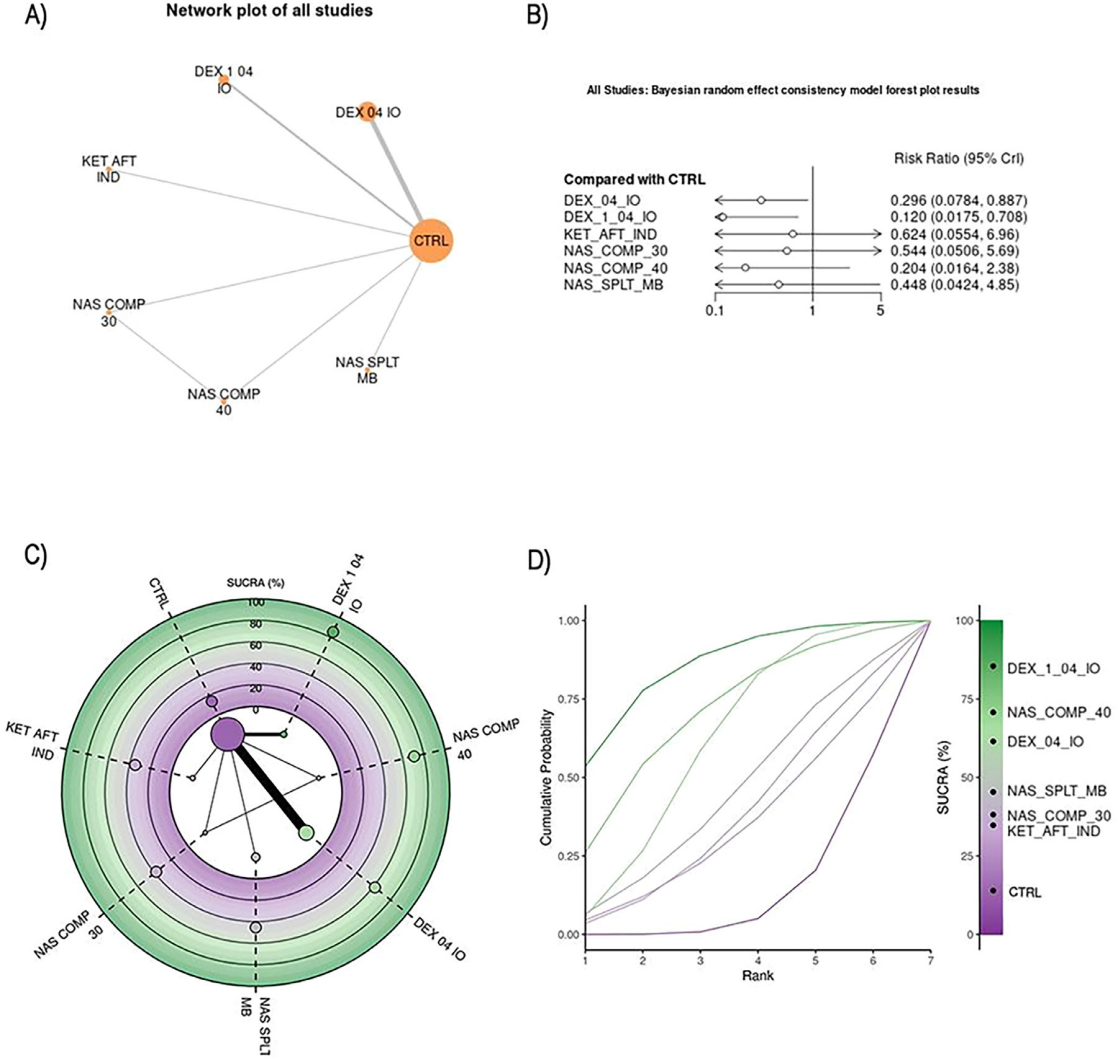
(A) A network plot of the studies included in the analysis that used sevoflurane for anesthesia maintenance. The number of studies that examined a treatment and compared two given treatments is represented by the size of the nodes and the thickness of the edges, respectively. (B) Bayesian random effect consistency model forest plot results. Forest plot for pairwise comparisons of included interventions versus placebo of the studies included in the analysis that used sevoflurane for anesthesia maintenance. The effect size is expressed as risk ratio. (C) Radial Surface Under the Cumulative Ranking Curve (SUCRA) plot of the studies included in the analysis that used sevoflurane for anesthesia maintenance. The treatments are ordered clockwise starting at “12 o'clock,” with the SUCRA value of each treatment plotted radially. The more outward the representation of the intervention, the higher its relative efficacy. The radial SUCRA contains a network of interventions with node sizes proportional to the evidence supporting them. (D) Litmus Rank-O-Gram in relation to individual outcomes of the studies included in the analysis that used sevoflurane for anesthesia maintenance. It shows the cumulative probability for each guidance technique to be ranked as first, second, third and beyond alongside with the SUCRA values. The higher the bar, the greater the probability of ranking in that position. CTRL, Control (Saline, placebo, or procedure not performed); DEX_04_IO, Intraoperative Dexmedetomidine infusion of 0.4 mcg.kg^-1^.h^-1^; DEX_1_0.4_IO, Intraoperative Dexmedetomidine bolus of 1 mcg.kg^-1^ for 10 min followed by infusion of 0.4 mcg.kg^-1^.h^-1^; KET_AFT_IND, Ketamine bolus of 1 mg.kg^-1^ after induction; NAS_COMP_30, Nasal compression for 30 min before induction of anesthesia; NAS_COMP_40, Nasal compression for 40 min before induction of anesthesia; NAS_SPLT_MB, Nasal splinting and mouth breathing training before procedure.

We chose to describe only the five best-ranked treatments in the overall analysis (Fig. 4). The ranking based on Surface Under the Cumulative Ranking (SUCRA) scores provides a comprehensive measure of the effectiveness of each intervention. SUCRA scores range from 0% to 100%, where a higher percentage indicates a higher probability of an intervention being among the best treatments. This ranking system considers the entire distribution of effect estimates, allowing for a more nuanced comparison of treatments. By incorporating the probability of each intervention occupying each rank, SUCRA scores offer a detailed understanding of how each treatment performs relative to others, beyond merely identifying statistical significance. Therefore, SUCRA scores in the ranking of treatments offers significant implications for evidence-based practice.

Credibility Intervals (CrIs) play a crucial role in interpreting the results of Bayesian analysis, offering a range within which the true effect size is likely to lie within a certain probability, in our study, 95%. The width of the credibility interval is influenced by the sample size and the number of studies analyzed; larger samples tend to yield narrower intervals, indicating greater precision in effect estimation. Narrow credibility intervals suggest that the estimated treatment effects are more reliable, whereas wider intervals indicate greater variability and uncertainty, suggesting that further research may be needed.

An intervention can be statistically significant within its confidence interval in a single study but not within its credibility interval when considering broader evidence due to the differences in how these intervals are constructed. Confidence intervals reflect the variability within the study's sample, indicating where the true effect is likely to fall in repeated sampling from the same population.

In contrast, credibility intervals incorporate prior knowledge, and the entire distribution of effect estimates across multiple studies. This means they account for more sources of variability and uncertainty. If an intervention's CrI is wide or includes the possibility of no effect, it suggests that, when considering all available evidence and prior beliefs, there is more uncertainty about the true effect size.

Almost all interventions analyzed were statistically significant when compared individually to their respective control groups in their respective studies, with the only exception being the article by Abitağaoğlu et al.^6^ The implication of an intervention being significant in its confidence intervals but not in its credibility intervals is that while the single study's result may be promising, the broader analysis incorporating more data and prior information indicates that the effect is less certain. This highlights the value of considering the full body of evidence to make more reliable clinical decisions.

#### Intraoperative dexmedetomidine^30^^,^^34^

Dexmedetomidine administered intraoperatively as a 1 mcg.kg^-1^ bolus over 10 minutes followed by an infusion of 0.4 mcg.kg^-1^.h^-1^ was ranked as the best intervention in both the overall analysis and the subgroup of non-sevoflurane inhalation anesthetics. This intervention showed a statistically significant effect in both the overall analysis (RR = 0.122, 95% CrIs: 0.0229 to 0.533) and the non-sevoflurane subgroup (RR = 0.120, 95% CrIs: 0.0175 to 0.708). The sevoflurane subgroup analysis could not be conducted due to the lack of studies evaluating dexmedetomidine in this specific context. The studies by Garg et al.^30^ and Naqvi et al.^34^ were reviewed, and no potential confounding factors affecting the estimated effect were identified.

Our analysis supports the evidence for using intraoperative dexmedetomidine infusion with a bolus for preventing emergence agitation in patients undergoing nasal surgery under inhalation anesthesia.

#### Bilateral nasociliary and maxillary nerve blocks^9^

Bilateral nasociliary and maxillary nerve block after general anesthesia was ranked as the second-best intervention for preventing emergence agitation in the overall analysis and the best intervention in the sevoflurane subgroup analysis. The intervention showed a statistically significant effect in the overall analysis (RR = 0.0114, 95% CrIs: 0.0125 to 0.898), but it was not statistically significant in the sevoflurane subgroup analysis (RR = 0.116, 95% CrIs: 0.00808 to 1.43). The non-sevoflurane subgroup analysis could not be conducted due to the absence of studies evaluating bilateral nasociliary and maxillary nerve blocks in this specific context. The study by Parthasarathy et al.^9^ was reviewed, and no potential confounding factors affecting the estimated effect were identified.

Despite the lack of statistical significance in the subgroup analysis for the credibility interval, our overall analysis demonstrates significant evidence supporting bilateral nasociliary and maxillary nerve blocks for preventing emergence agitation in patients undergoing nasal surgery under inhalation anesthesia.

#### Intraoperative ketamine^7^

Ketamine 0.5 mg.kg^-1^ 20 minutes before the end of surgery was ranked as the third-best intervention for preventing emergence agitation in the overall analysis and the second-best intervention in the sevoflurane subgroup analysis. However, it was not statistically significant in either analysis (overall: RR = 0.149, 95% CrIs: 0.0193 to 1.05; sevoflurane: RR = 0.149, 95% CrIs: 0.0121 to 1.75). The non-sevoflurane subgroup analysis could not be performed due to the absence of studies involving intraoperative ketamine in that setting.

The study by Demir et al.^7^ reported a statistically significant difference in anesthesia duration, with a longer anesthesia duration in the control group (113.62±9.80) compared to the intervention group (106.31±10.67). This discrepancy suggests a potential influence on the estimated effect of ketamine bolus 20 minutes before the end of surgery, possibly leading to an overestimation of its beneficial effects. Prolonged surgery durations have been identified as a risk factor for emergence agitation, raising the possibility that the higher incidence of emergence agitation could be attributed to extended exposure to anesthetics.^37^

Our analysis suggests that further research is needed to confirm the effectiveness of ketamine bolus 20 minutes before the end of surgery.

#### Nasal compression^14^

Nasal compression for 40 minutes before the induction of anesthesia was ranked as the fourth-best intervention for preventing emergence agitation in the overall analysis and the second-best intervention in the non-sevoflurane subgroup analysis. However, it was not statistically significant in either analysis (overall: RR = 0.207, 95% CrIs: 0.0244 to 1.61; non-sevoflurane: RR = 0.204, 95% CrIs: 0.0164 to 2.38). The sevoflurane subgroup analysis could not be performed due to the absence of studies evaluating nasal compression in that setting. The study by Kumari and Agrawal^14^ was reviewed, and no potential confounding factors affecting the estimated effect were identified.

Our analysis suggests that further research is needed to confirm the effectiveness of nasal compression for preventing emergence agitation.

#### Suction above cuff endotracheal tube^12^

Suction above the cuff of the endotracheal tube was ranked as the fifth-best intervention for preventing emergence agitation in the overall analysis and the third-best in the sevoflurane subgroup analysis. However, it was not statistically significant in either analysis (overall: RR = 0.252, 95% CrIs: 0.0244 to 2.09; sevoflurane: RR = 0.245, 95% CrIs: 0.0163 to 3.37). The non-sevoflurane subgroup analysis could not be performed due to the absence of studies evaluating suction above the cuff of the endotracheal tube in that setting. The study by Yuzkat et al.^12^ was reviewed, and no potential confounding factors affecting the estimated effect were identified.

Our analysis suggests that further research is needed to confirm the effectiveness of suction above the cuff endotracheal tube.

### Sensitivity analysis

There were no zero-event arms to perform a sensitivity analysis or enough plots to perform a nodesplit analysis.

### Risk of bias

**Figure 5:**
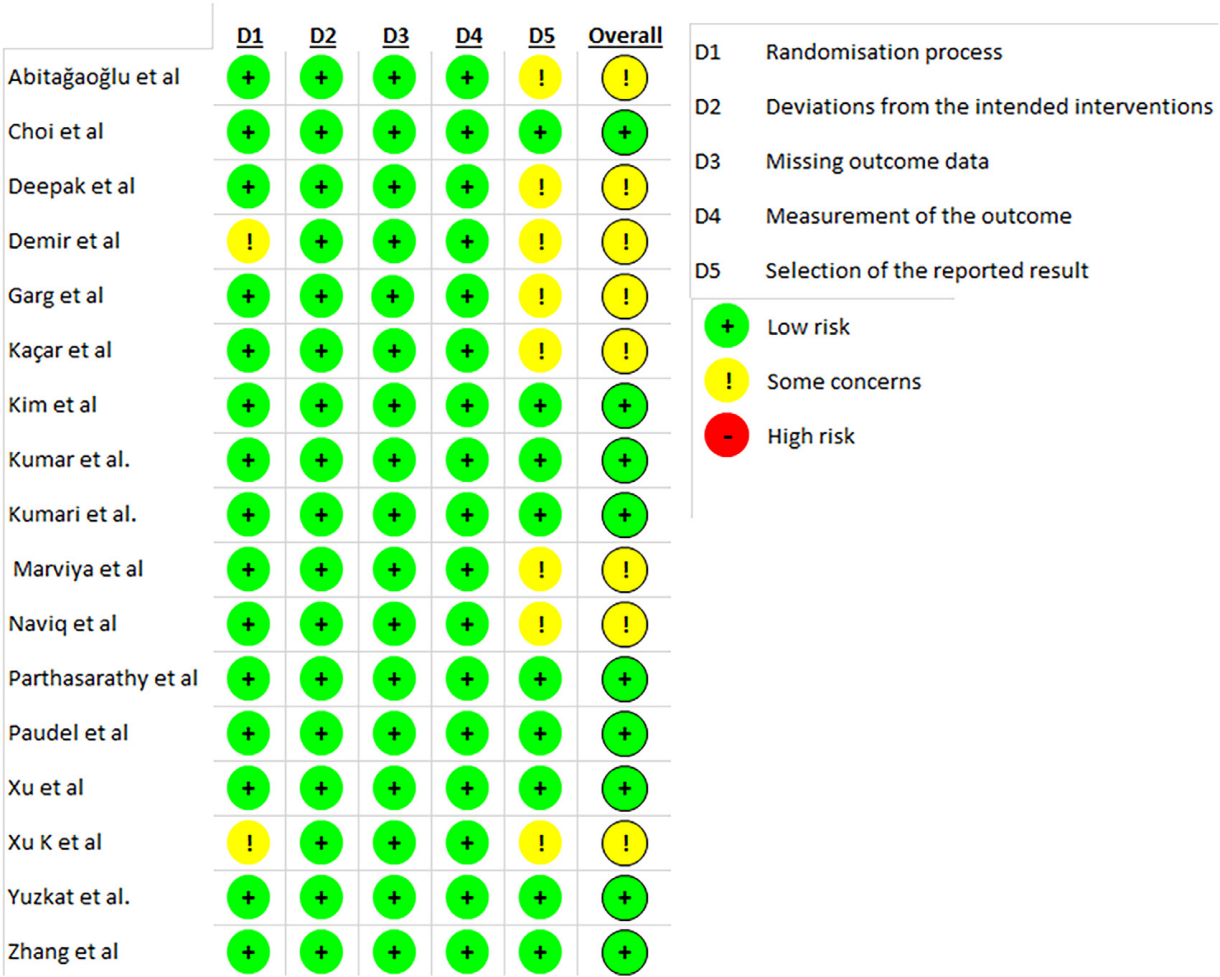
Risk of bias summary of included trials: evaluation of bias risk items for each included study. D1, randomization process; D2, deviations from intended interventions; D3, missing outcome data; D4, measurement of the outcome; D5, selection of the reported result. Green circle, low risk of bias; yellow circle, some concerns about bias; red circle, high risk of bias.

The risk of bias of the randomized controlled trial was summarized in Figure 5. Of the 17 trials, eight were graded as having some concerns of bias and nine as having a low risk of bias.

## Discussion

Our findings suggest that the most effective interventions for preventing emergence agitation include intraoperative dexmedetomidine bolus followed by infusion with the doses of 1 mcg.kg^-1^ for 10 minutes followed by 0.4 mcg.kg^-1^.h^-1^, bilateral nasociliary and maxillary nerve blocks, ketamine infusion at a dose of 0.5 mg.kg^-1^ 20 minutes before the end of the procedure, and non-pharmacological interventions such as nasal compression for 40 minutes before surgery and suction above the cuff endotracheal tube.

Although suction above the cuff endotracheal tube and ketamine infusion at the end of the procedure were highly ranked, their effectiveness was not confirmed by the credibility interval. Additionally, dexmedetomidine infusion at 0.4 mcg.kg^-1^.h^-1^ was statistically significant and ranked second in the subgroup analysis using non-sevoflurane inhalation agents for anesthesia maintenance but ranked seventh in the overall analysis.

Thus, different doses of dexmedetomidine, various timings of ketamine administration, different types of regional anesthetic blocks, and a range of non-pharmacological interventions were analyzed, necessitating a discussion on the implications of our findings for these strategies.

### Dexmedetomidine

Dexmedetomidine bolus followed by infusion was found to be the most effective intervention for preventing emergence agitation among the different methods of dexmedetomidine utilization. Studies employing similar doses of dexmedetomidine, whether through isolated bolus or infusion at the usual dose, yielded outcomes that were comparable to each other but inferior to the combined infusion and bolus administration. Future trials investigating the use of dexmedetomidine for emergence agitation prevention should consider this finding.

### Ketamine

Administering ketamine at 0.5 mg.kg^-1^ 20 minutes before the end of the procedure was ranked higher than administering ketamine at 1 mg.kg^-1^ 10 minutes after anesthetic induction. This observation suggests that the enhanced effectiveness of ketamine is likely attributable to its pharmacological activity during the immediate postoperative period or at the point of anesthetic emergence.

### Regional blocks

Performing nasociliary and maxillary nerve blocks after anesthesia induction was ranked higher than bilateral infraorbital and infratrochlear nerve blocks 30 minutes before surgery and preoperative bilateral infraoptic nerve and infratrochlear nerve blocks. It is unclear whether the superior effectiveness of nasociliary and maxillary nerve blocks is related to the type of regional blockade or to the timing of the blockade. Further studies should explore different regional block strategies and timings to optimize this intervention for emergence agitation prevention.

### Non-pharmacological interventions

Two main forms of non-pharmacological interventions were evaluated: techniques to educate patients on breathing through the mouth to reduce the sensation of suffocation associated with nasal surgeries and interventions aimed at reducing secretion impact during extubation.

Nasal compression for 40 minutes before anesthesia induction was the highest-ranked intervention and the only statistically significant one in the first group. Suction above the cuff endotracheal tube was the only intervention evaluated in the second group, showing a favorable ranking but with non-statistically significant results. These non-pharmacological strategies are relevant for anesthetic practice, as they are low-cost and easily implementable.

#### Diagnosis of emergence agitation

The diagnosis of emergence agitation in our meta-analysis relied on the Riker Agitation-Sedation Scale (SAS) and the Richmond Agitation-Sedation Scale (RASS).^38^, ^39^, ^40^, ^41^ Both scales are standardized and validated clinical tools with high interrater reliability and reproducibility. The SAS employs a numerical scale ranging from unarousable (score of 1) to dangerous agitation (score of 7) to assess sedation levels, while the RASS is a 10-point scale spanning from unarousable (score of -5) to combative (score of +4). Despite some differences in scoring, both scales share similar descriptions, with scores of 5 on the SAS and +1 on the RASS indicating the initial onset of agitation or anxiety.

For the identification of emergence agitation, the selected studies utilized the criteria of SAS ≥ 5 or RASS ≥ +1. However, it is noteworthy that two studies employing the SAS criteria did not specify the cutoff value used.^12^^,^^34^ Although previous research has demonstrated a high level of agreement between the two scales for assessing agitation,^42^ our meta-analysis is vulnerable to the implications arising from disparities in assessment methodologies between the scales, thereby constituting a limitation of our study.

### Limitations

Although our network meta-analysis has aggregated several valuable studies on the prevention of emergency agitation after nasal surgeries, its conclusions are subject to some limitations. Firstly, there was high heterogeneity among the included studies, and the number of patients undergoing each assessed intervention was restricted. Additionally, the included studies used two different scales for measuring emergency agitation; despite the scales demonstrating similar sensitivities and our exclusion of articles with excessively or insufficiently sensitive cutting points, there is a persistent possibility of bias in our results. Finally, even though the bias risk assessment for each included item has suggested a low or moderate risk, potential biases within the studies, along with the assumption of equivalence for different forms of control, may influence our findings.

## Conclusion

The present network meta-analysis, which included 2122 patients across 17 randomized controlled trials, identified effective strategies for enhancing perioperative care in nasal surgeries. Specifically, intraoperative dexmedetomidine infusion with bolus, bilateral nasociliary and maxillary nerve blocks administered after general anesthesia, and nasal compression for 40 minutes prior to induction were associated with statistically significant reductions in the risk of emergence agitation. These findings provide robust evidence for tailoring perioperative management strategies based on emergence agitation outcomes. Implementing these measures could significantly improve patient outcomes and enhance overall perioperative care.

## Authors' contributions

The authors confirm contribution to the paper as follows: study conception and design: Gustavo R. M. Wegner, Gilberto Tadeu Ferrugem de Oliveira; data collection: Gustavo R. M. Wegner, Henrik G. Oliveira; analysis and interpretation of results: Gustavo R. M. Wegner, Bruno F. M. Wegner, Luis A. Costa, Luigi W. Spagnol, Valentine W. Spagnol; draft manuscript preparation: Gustavo R. M. Wegner, Gilberto Tadeu Ferrugem de Oliveira, Bruno F. M. Wegner, Henrik G. Oliveira, Luis A. Costa, Luigi W. Spagnol, Valentine W. Spagnol. All authors reviewed the results and approved the final version of the manuscript.

## Conflicts of interest

The authors declare no conflicts of interest.

## Appendix Supplementary materials

Image, application 1

## Footnotes

Supplementary material associated with this article can be found in the online version at doi:10.1016/j.bjane.2024.844565.
