# Supplementary material for: Pharmacological and non-pharmacological interventions in patients undergoing nasal surgeries for prevention of emergence agitation: a systematic review and network meta-analysis
Source: Braz J Anesthesiol. 2024 Oct 16;75(1):844565. doi: 10.1016/j.bjane.2024.844565 (PMC11555333; doi:10.1016/j.bjane.2024.844565)

BJAN-D-24-00145_Supplementary Material

**Table S1** Characteristics of included studies.

| **Author** | **Year** | **Local** | **Type of surgery** | **n** | **Age** | **Male (%)** | **Arm 1/ Arm 2 / Arm 3** |
| --- | --- | --- | --- | --- | --- | --- | --- |
| Abitağaoğlu et al. | 2021 | Turkey | Septoplasty | 102 | 33.54 [10.71] | 71.15 | Ketamine 1 mg.kg^-1^ IV in 10 mins after anesthesia induction/Saline used instead of ketamine |
|  |  |  |  |  | 32.80 [10.35] | 64.00 |  |
| Choi et al. | 2019 | Korea | OSRP | 66 | 21.97 [1.47] | 100 | Bilateral infraorbital and infratrochlear nerve block with 8 mL of 0.5% ropivacaine 30 mins prior to surgery/Saline used instead of ropivacaine |
|  |  |  |  |  | 22.38 [3.0] | 100 |  |
| Deepak et al. | 2018 | India | FESS | 56 | 38.30 [11.70] | 89.28 | Continuous infusion of Dexmedetomidine at 0.4 mcg.kg^-1^.h^-1^ from induction of anesthesia until nasal packing/Volume matched saline infusion |
|  |  |  | Septoplasty |  | 35.50 [11.80] | 75.00 |  |
| Demir et al. | 2018 | Turkey | Rhinoplasty | 140 | 25.80 [7.45] | 47.14 | Ketamine 0.5 mg.kg^-1^ IV applied 20 minutes before the end of surgery/Saline applied instead of ketamine |
|  |  |  |  |  | 24.88 [4.78] | 48.57 |  |
| Garg et al. | 2018 | India | FESS | 72 | 28.61 [11.72] | 66.66 | Dexmedetomidine infusion 1 mcg.kg^-1^ for 10 min as a bolus than 0.4 mcg.kg^-1^.h^-1^ after intubation /0.9% normal saline bolus for 10 min and then maintenance infusion after intubation |
|  |  |  | Septoplasty |  | 30.89 [13.54] | 58.33 |  |
|  |  |  | Dacryocystorhinostomy |  |  |  |  |
|  |  |  | Adenoidectomy |  |  |  |  |
|  |  |  | Nasal bone fracture |  |  |  |  |
| Kaçar et al. | 2020 | Turkey | Septorhinoplasty | 52 | 27.38 [7.09] | 42.30 | Preoperative bilateral infraoptic nerve and infratrochlear nerve blocks using a 4 mL injection of 0.5% bupivacaine with a 25-gauge needle / Not performed |
|  |  |  |  |  | 24.50 [5.50] | 50.00 |  |
| Kim et al. | 2013 | Korea | Septoplasty / Septoplasty + Ethmoidectomy / Ethmoidectomy / Septorhinoplasty | 100 | 32 (20–58) | 74.00 | Intraoperative continuous dexmedetomidine infusion 0.4 mcg.kg^-1^.h^-1^ until extubation / Volume matched normal saline |
|  |  |  |  |  | 33 (20–58) | 70.00 |  |
| Kumar | 2020 | India | Not specified | 100 | 33.32 [10.18] | 78.00 | Intraoperative continuous dexmedetomidine infusion at a rate of 0.4 mcg.kg^-1^.h^-1^ until extubation / Volume matched normal saline |
|  |  |  |  |  | 36.92 [11.61] | 82.00 |  |
| Kumari et al. | 2023 | India | Rhinoplasty | 75 | 28.6 [3.2] | 44.00 | Nasal compression for 30 minutes before induction of anesthesia / Nasal compression for 40 minutes before induction of anesthesia / No nasal compression before induction of anesthesia. |
|  |  |  | Septoplasty |  | 29.2 [2.4] | 52.00 |  |
|  |  |  | Septorhinoplasty |  | 28.7 [3.5] | 56.00 |  |
|  |  |  | Turbinoplasty |  |  |  |  |
|  |  |  | Functional Endoscopic Sinus Surgery |  |  |  |  |
| Marviya et al. | 2019 | India | Not specified | 70 | 33.14 [12.56] | 65.71 | Dexmedetomidine 2 mcg.mL^-1^ at rate of 0.4 mcg.kg^-1^.hr^-1^ was started after induction of anesthesia & stopped after extubation / Volume matched normal saline |
|  |  |  |  |  | 33.25 [10.89] | 68.57 |  |
| Navqi et al. | 2021 | Pakistan | FESS | 60 | 28.10 [5.58] | 46.66 | 1 mcg.kg^-1^ dexmedetomidine infusion for 10 minutes and 0.4 mcg.kg^-1^.h^-1^ maintenance dose as infusion following tracheal intubation / Volume matched saline |
|  |  |  | DCR |  | 29.83 [4.62] | 53.33 |  |
|  |  |  | Septoplasty Adenoidectomy |  |  |  |  |
| Parthasarathy et al. | 2022 | India | FESS | 51 | 36.73 [8.92] | 80.76 | Bilateral nasociliary and maxillary nerve blocks with 12 mL of equal volumes of 0.5% bupivacaine and 2% lignocaine after induction of general anesthesia / No nerve block |
|  |  |  | Septoplasty |  | 35.08 [11.24] | 60.00 |  |
|  |  |  | FESS + Polypectomy |  |  |  |  |
| Paudel et al. | 2024 | Nepal | Not specified | 100 | 37.2 [11.04] | 74.00 | Dexmedetomidine at a rate of 0.4 µg.kg^-1^.h^-1^ after induction of anesthesia till extubation / Volume matched normal saline |
|  |  |  |  |  | 37.5 [13.15] | 70.00 |  |
| Xu et al. | 2023 | China | FESS | 182 | 51.00 [20.78] | 59.78 | Nasal splint preconditioning and mouth breath training in pre-anaesthesia room 1h before surgery. /No intervention |
|  |  |  |  |  | 53.00 [18.82] | 60.00 |  |
| Xu K et al. | 2016 | China | Endoscopic sinus surgery | 60 | 37.5 [12.30] | 63.33 | Intravenous administration of dexmedetomidine 0.5 mcg.kg^-1^ after endotracheal intubation / Volume matched normal saline |
|  |  |  |  |  | 40.2 [11.50] | 70.00 |  |
| Yuzkat et al. | 2019 | Turkey | Septorhinoplasty | 132 | 26.30 [5.80] | 43.93 | Suction Above Cuff Endotracheal Tube / Classic endotracheal tube |
|  |  |  |  |  | 25.60 [7.70] | 39.39 |  |
| Zhang et al. | 2023 | China | FESS | 708 | 51 (18‒65) | 62.29 | Butorphanol infusion 20 mcg.kg^-1^ before anesthesia induction / Volume matched saline |
|  |  |  |  |  | 51 (18‒65) | 60.28 |  |

**Table S2** Characteristics of included studies.

| **Author** | **Anesthesia maintenance / Analgesia** | **Inclusion criteria** | **Exclusion criteria** | **Emergence delirium assessment** |
| --- | --- | --- | --- | --- |
| Abitağaoğlu et al. | Mixture of 50% oxygen and 5%–7% desflurane in air at a flow rate of 2 L.min^-1^. / Remifentanil infusion at a rate of 0.05–2 mcg.kg^-1^. min^-1^ | 1. ASA I‒II  2. Adult patients  3. Scheduled to septoplasty | 1. Ketamine allergy  2. Hypertension  3. Coronary artery disease  4. Arrhythmia  5. Seizure  6. Obstructive sleep apnea  7. Glaucoma  8. Kidney disease  9. Liver disease  10. BMI > 30  11. Cognitive dysfunction | Riker Sedation/Agitation Scale (SAS)  - SAS ≥ 5 considered agitated  - The period between the discontinuation of inhalation anesthesia and 3 minutes after extubation was considered the emergence period. |
| Choi et al. | Sevoflurane (1.0–1.5 age-adjusted minimal alveolar concentration) / Remifentanil infusion at a rate of 0.05–0.2 mcg.kg^-1^ min^-1^ | 1. ASA I‒II  2. Patients aged 18‒65  3. Scheduled for septorhinoplasty | 1. ASA ≥ 3  2. Local anesthetic drugs allergies  3. Preoperative chronic pain  4. Proven coagulopathy  5. Inability to provide informed consent  6. BMI > 35 | Riker Sedation/Agitation Scale (SAS)  - SAS ≥ 5 considered agitated  - The patients were continuously monitored from the time of extubation until discharge from the PACU by the investigator, who recorded the SAS score whenever it changed |
| Deepak et al. | Isoflurane (1‒1.2 minimal alveolar concentration), 66% N2O, 33% O2 / – | 1. ASA I‒II  2. Patients aged 18‒60  3. Scheduled for elective nasal surgeries  4. Nasal packing on each side was used for 24 hours | 1. Allergy to α-2 receptor agonist  2. Allergy to NSAIDS  3. Uncontrolled hypertension  4. Heart block greater than 1^st^ degree  5. Using MAO inhibitors  6. Using adrenergic blocking drugs  7. Cognitive impairment  8. Chronic use of antipsychotic medications  9. Alcohol abuse  10. Clinically significant neurologic disease  11. Clinically significant cardiovascular disease  12. Clinically significant renal disease  13. Clinically significant hepatic disease  14. Clinically significant gastrointestinal disease  15. Requiring surgeries longer than 2 hours duration | Riker Sedation/Agitation Scale (SAS)  - SAS ≥ 5 considered agitated  - SAS assessed at time of awakening or just before extubation and at 5 minutes after extubation |
| Demir et al. | 40% O2 and sevoflurane at 1.5% volume. / l mcg.kg^-1^ fentanyl applied at 30 mins intervals | 1. ASA I‒II  2. Adult patients  3. Scheduled for elective rhinoplasty | 1. Patients under 18 years of age  2. Patients who got pain relievers or sedatives in the last 24 hours before operation  3. History of neurological diseases  4. History of cardiological diseases  5. History of psychiatric diseases  6. ASA ≥ 3  7. Mentally retarded patients  8. Patients who consumed alcohol  9. History of previous rhinoplasty | Richmond Agitation–Sedation Scale (RASS):  - RASS ≥ 1 at any time were considered as agitated  - Assessed just after extubation and on admission to the PACU |
| Garg et al. | Desflurane was started at 6% dial flow concentration with 50:50 air and oxygen mixture / Fentanyl 1 mcg.kg^-1^ IV used intermittently | 1. ASA I‒II  2. Patients aged 18‒65  3. Scheduled for elective nasal surgeries of more than 1 hour duration under desflurane anaesthesia | 1. Cardiac disease  2. Hepatic disease  3. Renal disease  4. Pulmonary disease  5. Endocrinal disease  6. Neurological disease  7. Psychiatric disease  8. Substance abuse disorder  9. BMI > 35  10. On medication (beta-blockers, α2-agonists, opioids, clonidine, and tricyclic antidepressant)  11. Known allergy to study drugs  12. Pregnant/breast feeding females | Riker Sedation/Agitation Scale (SAS)  - SAS ≥ 5 considered agitated  - Emergence time was defined as the time interval from ‘T0’ till the patient was shifted to PACU once he/she tells his/her name.  T0 = “Desflurane and study drug was stopped when surgical dressing was applied, and the time (T0) was noted” |
| Kaçar et al. | 1.5–2 end-tidal minimal alveolar concentration value of sevoflurane / Remifentanil infusion at a rate of 0.05–0.2 mcg.kg^-^ min^-^ | 1. ASA I‒II  2. Patients aged 18‒65  3. Scheduled for septorhinoplasty | 1. Pregnancy  2. Cancer  3. Known local anesthetic drug allergy  4. Preoperative chronic pain  5. Inability to provide an informed consent  6. BMI > 35  7. Coagulopathy | Riker Sedation/Agitation Scale (SAS)  - SAS ≥ 5 considered agitated  - During the period from extubation to the discharge from postanaesthesia care unit (PACU), patients agitation status was evaluated using the Riker Sedation Agitation Scale |
| Kim et al. | Desflurane, which was regulated 0.6–1.4 age-adjusted minimal alveolar concentration (MAC) / – | 1. ASA I‒II  2. Patients aged 20‒58  3. Scheduled for elective nasal surgery  4. Nasal packing on each side was used until 24h after surgery | 1. Known or suspected allergy to α2-adrenergic agonist  2. Known or suspected allergy non-steroidal anti-inflammatory drugs.  3. Use of monoamine oxidase inhibitors or adrenergic blocking drugs.  4. History of uncontrolled hypertension.  5. Heart block greater than first degree.  6. Cognitive impairment. 7. Chronic use of antipsychotic medications.  8. Kidney disease  9. Liver disease.  10. BMI ≥ 30 | Riker Sedation/Agitation Scale (SAS)  - SAS ≥ 5 considered agitated  - Emergence is defined as the time interval from ‘time zero’ to 2 min after extubation |
| Kumar | 1 MAC desflurane in 50% air/oxygen mixture. The attending anesthesiologist was free to administer additional doses of fentanyl as he/she may feel appropriate. | 1. Age 20‒60 years.  2. ASA 1‒2  3. Patients undergoing elective nasal surgeries under general anesthesia and requiring nasal packing after surgery. | 1. Known or suspected allergy to α2 adrenergic agonists.  2. Use of monoamine oxidase inhibitors, adrenergic blocking agents or clonidine.  3. Uncontrolled hypertension and Diabetes mellitus.  4. Heart block greater than first degree.  5. Cognitive impairment.  6. Chronic use of antipsychotic medications.  7. Kidney or liver disease.  8. Body mass index ≥ 30 Kg.m^-2^.  9. Pregnancy. | Riker Sedation/Agitation Scale (SAS)  - SAS ≥ 5 considered agitated  - Emergence is defined as the time interval from ‘time zero’ to 2 min after extubation |
| Kumari et al. | Anesthesia was maintained with a mixture of oxygen and nitrous oxide (50:50), isoflurane (1%‒1.5%) | 1. ASA 1‒2  2. Age 18‒65 years  3. Elective nasal surgeries under general anesthesia with postoperative bilateral nasal packing | 1. Hepatic, renal, cardiac, or respiratory comorbidities  2. Bleeding disorders or coagulopathies  3. History of smoking  4. Body Mass Index (BMI) ≥ 30 kg/m^2^  5. History of snoring and obstructive sleep apnea  6. Prolonged laryngoscopy and intubation duration (> 3 minutes)  7. Requirement of multiple intubation attempts  8. Airway trauma caused during the procedure | Riker Sedation/Agitation Scale (SAS)  - SAS ≥ 5 considered agitated  - Emergence agitation (according to the RSAS) was assessed at extubation and then every five minutes for the first 15 minutes, and then every 15 minutes thereafter until discharge from the PACU |
| Marviya et al. | Oxygen, nitrous oxide, sevoflurane / – | 1. ASA 1‒2  2. Patients aged 20‒58  3. Scheduled for elective nasal surgery  4. Nasal packing on each side was used until 24h after surgery | 1. Known or suspected allergy to α2-adrenergic agonist  2. Known or suspected allergy non-steroidal anti-inflammatory drugs.  3. Use of MAO inhibitors or adrenergic blocking drugs  4. History of uncontrolled hypertension  5. Second or third degree heart block  6. Impaired cognition  7. Chronic use of antipsychotic medications  8. Renal failure  9. Liver disease  10. BMI ≥ 30 | Riker Sedation/Agitation Scale (SAS)  - SAS ≥ 5 considered agitated  - Emergence is defined as the time interval from ‘time zero’ to 2 min after extubation |
| Naqvi et al. | The starting dial flow of desflurane was 6% in air and oxygen mixture (50:50) and it was titrated to sustain BIS 45‒55 during the surgery | 1. ASA 1‒2  2. Patients aged 18‒60  3. Elective nasal surgery  4. General anesthesia with desflurane | 1. Systemic illnesses (such as cardiac, hepatic, endocrinal, or neurological conditions)  2. Substance-induced disorder  3. Psychiatric disorders  4. Use of medications such as alpha-2 agonists  5. Use of beta blockers  6. Use of tricyclic antidepressants | Riker Sedation/Agitation Scale (SAS)  - The time interval from T0till the patient could tell his/her name was defined as emergence time. |
| Parthasarathy et al. | Sevoflurane in oxygen and 66% nitrous oxide to achieve an end-tidal concentration equivalent to 1 minimum alveolar concentration. / – | 1. ASA I‒II  2. Patients aged 18‒60  3. Scheduled for elective nasal surgery | 1. Patients with allergy to local anesthetic solution.  2. Patients with bleeding disorders.  3. Pregnant patients.  4. Patients unable to comprehend the study protocol. | Riker Sedation/Agitation Scale (SAS)  - SAS ≥ 5 considered agitated  - Emergence agitation was assessed from the moment of spontaneous breathing to extubation and the ability to respond to verbal requests. |
| Paudel et al. | Isoflurane was utilized to keep the anesthetic at 1%‒1.5 volume % for maintenance of anesthesia. | 1. ASA 1‒2  2. Patients aged 20‒60  3. Undergoing nasal and pharyngeal surgery and surgery not lasting more than two hours. | 1. Unwilling to participate  2. Allergic to the study drug  3. Had coagulopathies | Riker Sedation/Agitation Scale (SAS)  - SAS ≥ 5 considered agitated |
| Xu et al. | Desflurane inhalation, and the minimum alveolar concentration (MAC) value was controlled between 0.8 and 1.0 / A dose of 5 or 10 µg sufentanil was administered depending on patient’s weight and circulation status before performing the surgery. | 1. ASA 1‒2  2. Patients aged over 18  3. Scheduled to undergo functional endoscopic sinus surgery  4. Normal preoperative liver and kidney function | 1. Severe cardiovascular disease  2. ASA III‒IV  4. Poor blood pressure control  5. History of mental illness  6. Neurological diseases  7. Usage of sedative or antipsychotic drug  8. Nasal malformation  9. History of nasal trauma or implantation of nasal prosthesis. | Riker Sedation/Agitation Scale (SAS)  - SAS ≥ 5 considered agitated  - Assessed 30 min after extubation |
| Xu K et al. | Inhalation of 2.0%‒3.0% sevoflurance in 50% oxygen/air / Remifentanil 0.1‒0.2 mcg kg^-1^ min^-1^ | 1. ASA 1‒2  2. Patients aged 20‒60  3. scheduled for endoscopic sinus surgery for chronic sinusitis | 1. Cardiovascular or respiratory dysfunction  2. Any kind of cardiac conduction disorder  3. History of hepatic insufficiency  4. Allergy to alfa-2 agonists  5. Chronic use of psychotropic medications | Riker Sedation/Agitation Scale (SAS)  - SAS ≥ 5 considered agitated  - Emergence was defined as the period from discontinuation of anesthetic to 5-min after extubation.  - Assessed in the emergence period |
| Yuzkat et al. | 1% sevoflurane, 40% oxygen, and 60% medical air were used. / When needed for a maintenance dose, 0.5 mcg.kg^-1^ fentanyl was used | 1. ASA 1‒2  2. Patients aged 18‒65  3. Scheduled for rhinoplasty | 1. Upper or lower respiratory tract infections.  2. Asthma.  3. History of allergies.  4. Received isoflurane or desflurane for maintenance of anesthesia.  5. ASA class III–IV.  6. Difficult airway (Mallampati score III–IV).  7. Long uvula.  8. Gastroesophageal reflux.  9. Electrolyte disturbances such as hypomagnesemia and hypocalcemia.  10. BMI > 30 | Riker Sedation/Agitation Scale (SAS)  - Not informed about when it was assessed or about the cutoff to be considered agitated |
| Zhang et al. | Sevoflurane based on 1.3 age-adjusted minimum alveolar concentration / Remifentanil 0.2–0.5 mcg.kg^-1^.min^-1^ | 1. ASA 1/2  2. Patients aged 18‒65  3. Scheduled for functional endoscopic sinus surgery | 1. BMI > 30  2. Cerebral disease or patients with a history of neurological and psychiatric diseases, including Alzheimer disease, stroke, epilepsy, and psychosis  3. Bradycardia (heart rate < 60 beats per minute for any reasons)  4. gastrointestinal ulcer  5. Urinary incontinence  6. Asthma or chronic obstructive pulmonary disease  7. Allergy to butorphanol  8. Auditory or vision disorders  9. Unwillingness to comply with the protocol or procedures  10. Inability to communicate in Chinese Mandarin. | Riker Sedation/Agitation Scale (SAS)  - SAS ≥ 5 considered agitated  - Emergence duration is defined as the time spent in the PACU.  - During emergence, the level of agitation was evaluated |

**Table S3** Model fit for Bayesian network meta-analysis (inhalation agents for anesthesia maintenance).

| **Dbar** | 36.146 |
| --- | --- |
| **pD** | 33.201 |
| **DIC** | 69.348 |
| **Data points** | 35.000 |

Between-study standard deviation (log probability scale): 0.76.

95% Credible Interval: 0.12, 1.93.

**Table S4** Characteristics of all studies in inhalation agents for anesthesia maintenance.

| **Characteristic** | **Value** |
| --- | --- |
| Number of Interventions | 14 |
| Number of Studies | 17 |
| Total Number of Patients in Network | 2122 |
| Total Possible Pairwise Comparisons | 91 |
| Total Number of Pairwise Comparisons with Direct Data | 14 |
| Is the network connected? | TRUE |
| Number of Two-arm Studies | 16 |
| Number of Multi-Arms Studies | 1 |
| Total Number of Events in Network | 643 |
| Number of Studies with No Zero Events | 17 |
| Number of Studies with At Least One Zero Event | 0 |
| Number of Studies with All Zero Events | 0 |

**Table S5** Model fit for Bayesian network meta-analysis (sevoflurane maintenance group).

| **Dbar** | 16.405 |
| --- | --- |
| **pD** | 15.926 |
| **DIC** | 32.331 |
| **Data points** | 16.000 |

Between-study standard deviation (log probability scale): 0.95.

95% Credible Interval: 0.04, 1.85.

**Table S6** Characteristics of all studies in sevoflurane maintenance group.

| **Characteristic** | **Value** |
| --- | --- |
| Number of Interventions | 9 |
| Number of Studies | 8 |
| Total Number of Patients in Network | 1275 |
| Total Possible Pairwise Comparisons | 36 |
| Total Number of Pairwise Comparisons with Direct Data | 8 |
| Is the network connected? | TRUE |
| Number of Two-arm Studies | 8 |
| Number of Multi-Arms Studies | 0 |
| Total Number of Events in Network | 379 |
| Number of Studies with No Zero Events | 8 |
| Number of Studies with At Least One Zero Event | 0 |
| Number of Studies with All Zero Events | 0 |

**Table S7** Model fit for Bayesian network meta-analysis (non-sevoflurane inhalation agents for anesthesia maintenance group).

| **Dbar** | 19.521 |
| --- | --- |
| **pD** | 17.901 |
| **DIC** | 37.422 |
| **Data points** | 19.000 |

Between-study standard deviation (log probability scale): 0.96.

95% Credible Interval: 0.22, 2.19.

**Table S8** Characteristics of all studies (non-sevoflurane inhalation agents for anesthesia maintenance group).

| **Characteristic** | **Value** |
| --- | --- |
| Number of Interventions | 7 |
| Number of Studies | 9 |
| Total Number of Patients in Network | 847 |
| Total Possible Pairwise Comparisons | 21 |
| Total Number of Pairwise Comparisons with Direct Data | 7 |
| Is the network connected? | TRUE |
| Number of Two-arm Studies | 8 |
| Number of Multi-Arms Studies | 1 |
| Total Number of Events in Network | 264 |
| Number of Studies with No Zero Events | 9 |
| Number of Studies with At Least One Zero Event | 0 |
| Number of Studies with All Zero Events | 0 |

**Figure S1** Deviance leverage plot (inhalation agents for anesthesia maintenance).


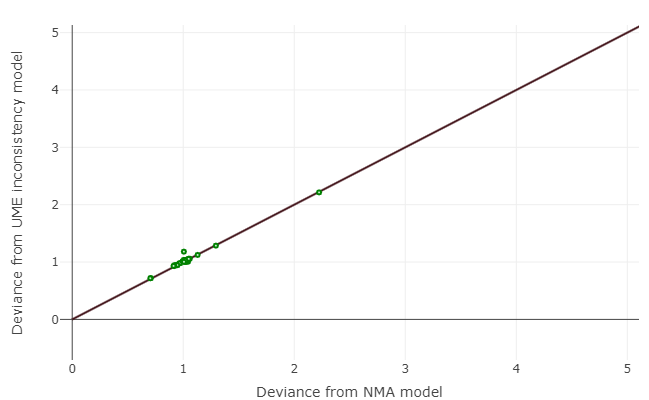


**Figure S2** Per-arm residual deviance for all studies in inhalation agents for anesthesia maintenance analysis.


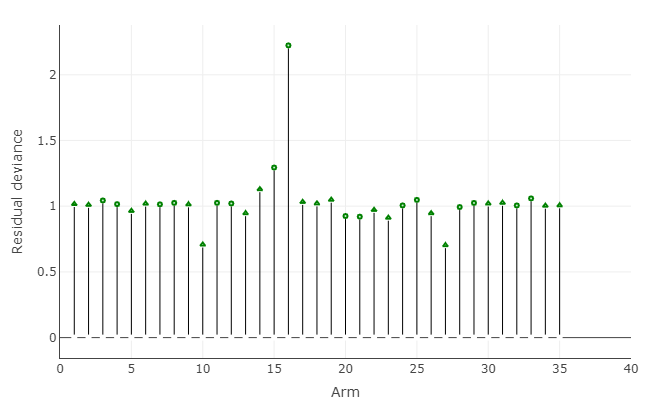


**Figure S3** Residual deviance from NMA model and UME inconsistency model for all studies in inhalation agents for anesthesia maintenance analysis.


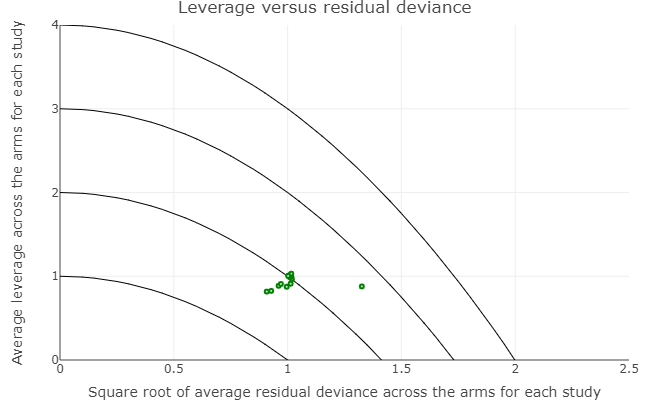


**Figure S4** Individual results for all studies in inhalation agents for anesthesia maintenance analysis grouped by comparison


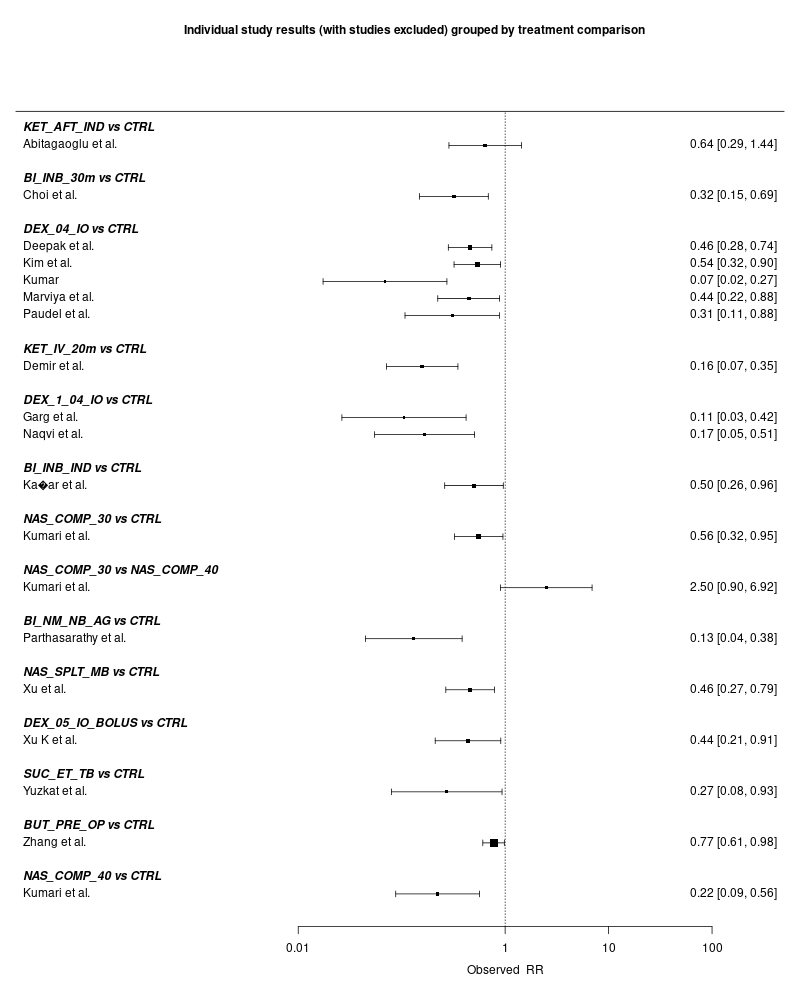


**Figure S5** Deviance leverage plot (sevoflurane maintenance group).


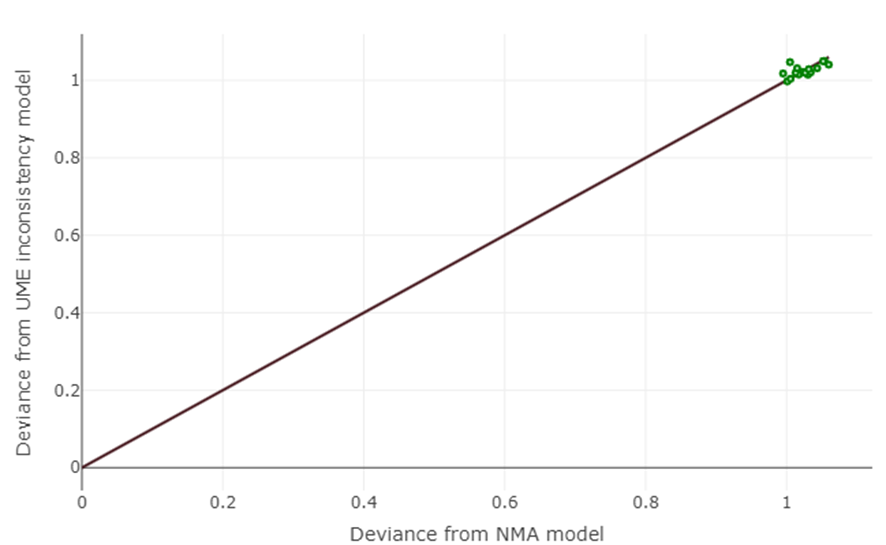


**Figure S6** Per-arm residual deviance for all studies in sevoflurane maintenance group.


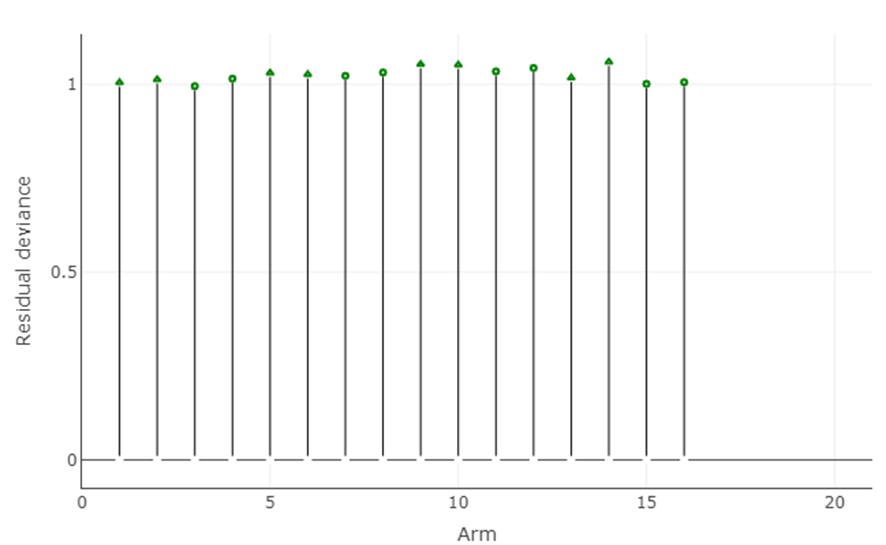


**Figure S7** Residual deviance from NMA model and UME inconsistency model for all studies in sevoflurane maintenance group.


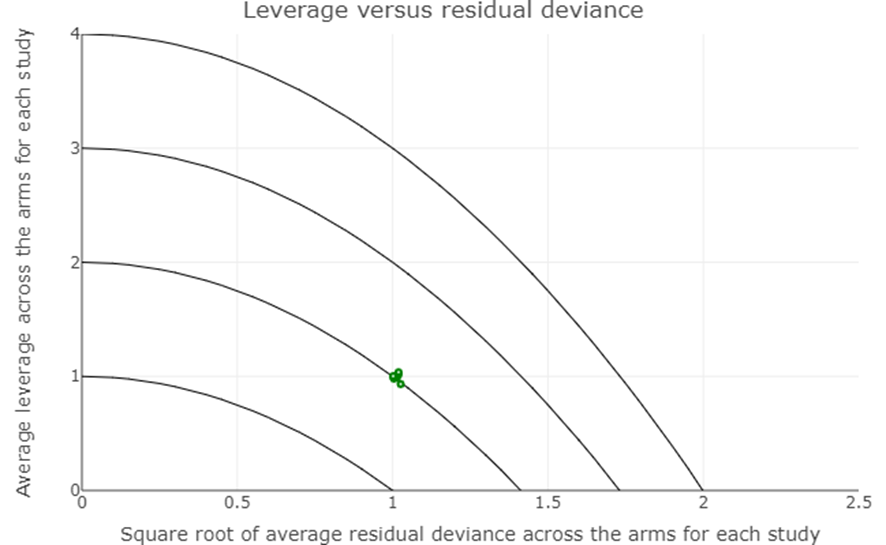


**Figure S8** Individual results for all studies in sevoflurane maintenance group grouped by comparison.


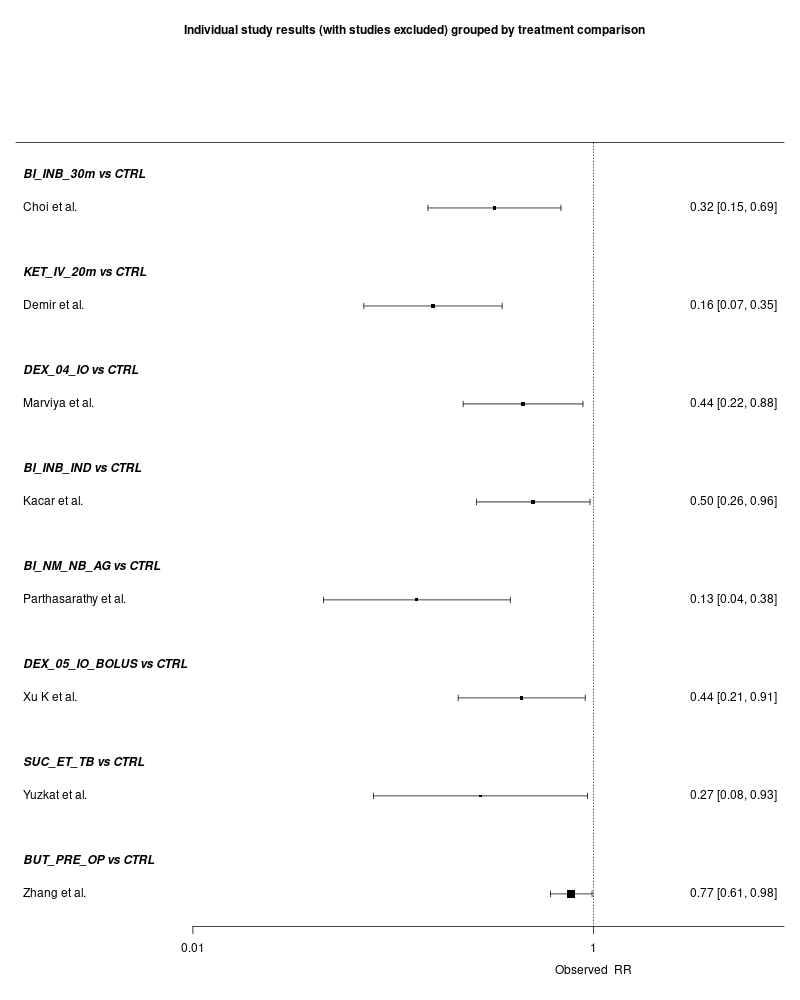


**Figure S9** Deviance leverage plot (non-sevoflurane maintenance group).


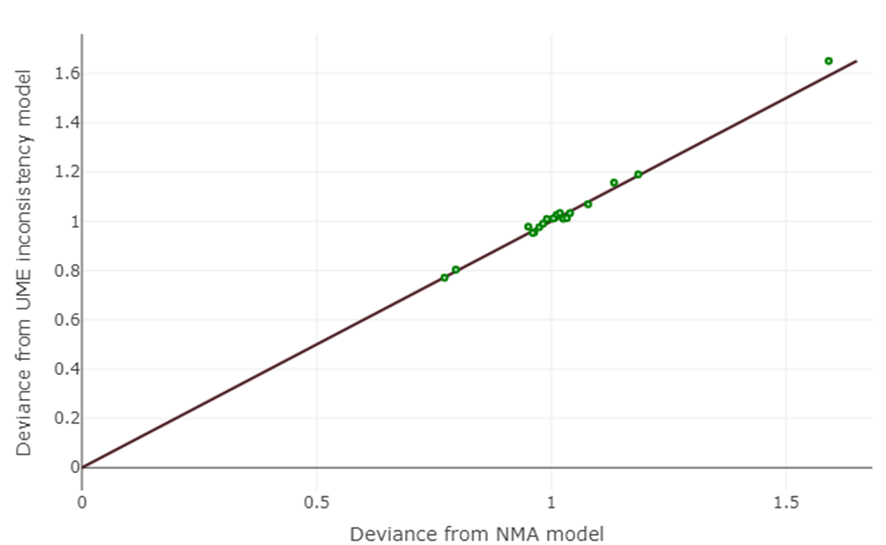


**Figure S10** Per-arm residual deviance for all studies in non-sevoflurane maintenance group.


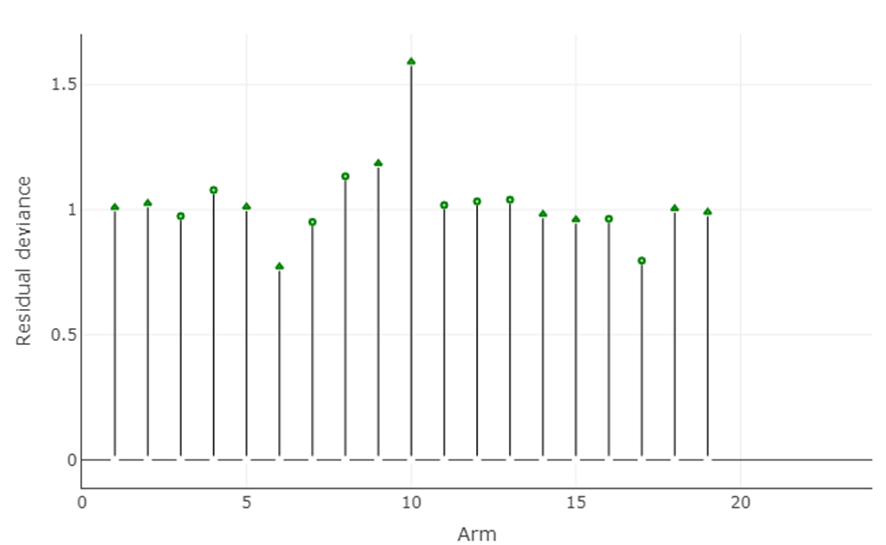


**Figure S11** Residual deviance from NMA model and UME inconsistency model for all studies in non-sevoflurane maintenance group.


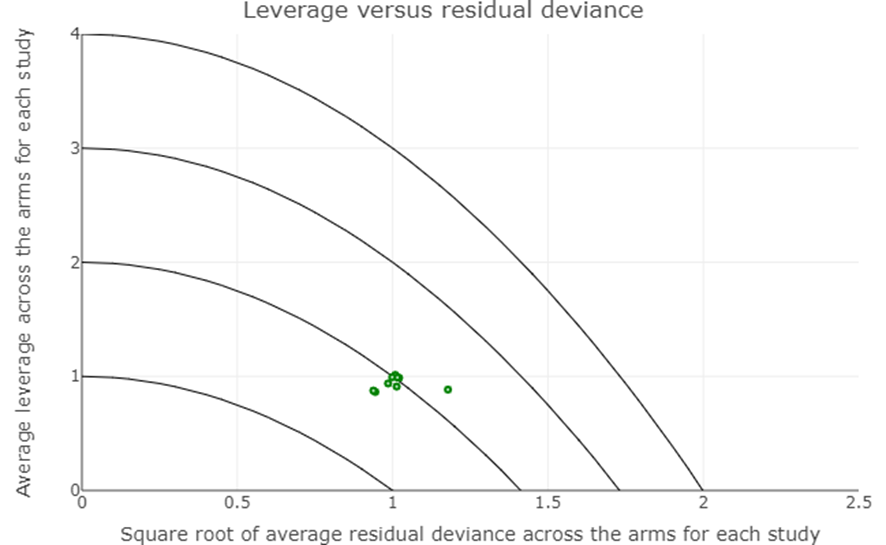


**Figure S12** Individual results for all studies in non-sevoflurane maintenance group grouped by comparison.


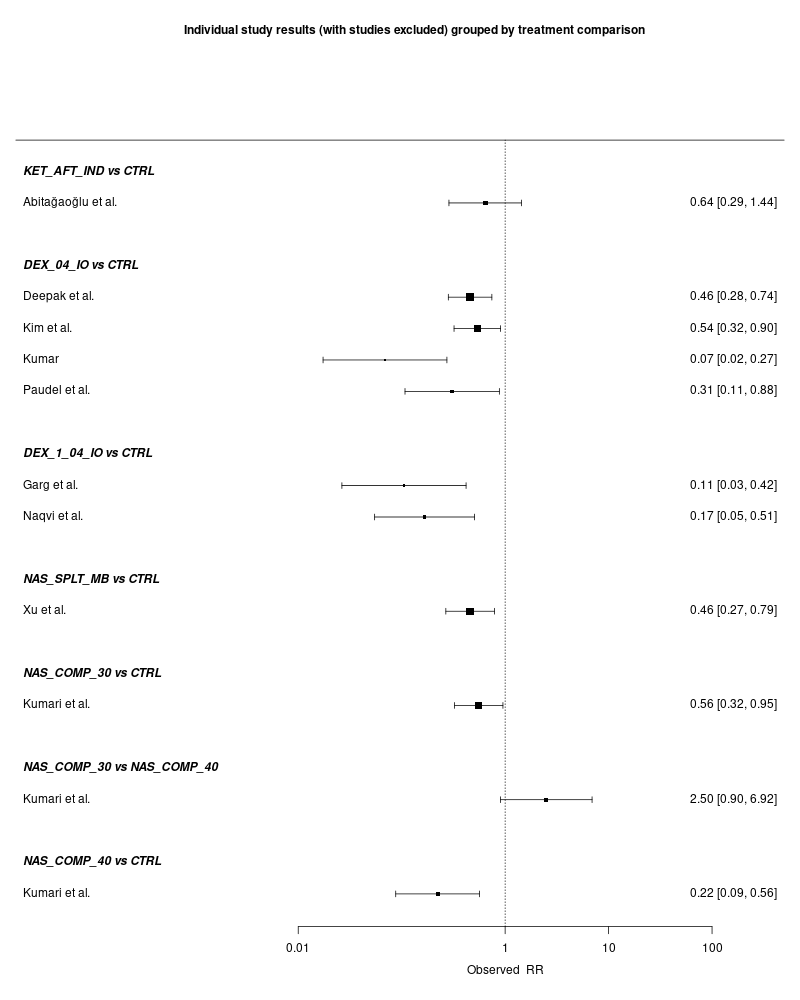


**Figure S13** Gelman convergence assessment plot for all studies in inhalation agents for anesthesia maintenance analysis.


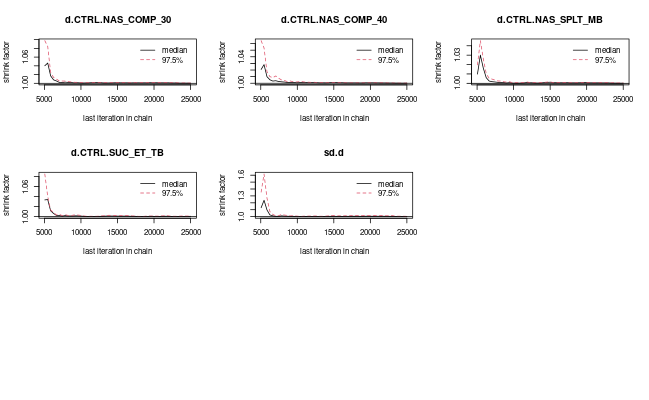


**Figure S14** Gelman convergence assessment plot for all studies in sevoflurane group.


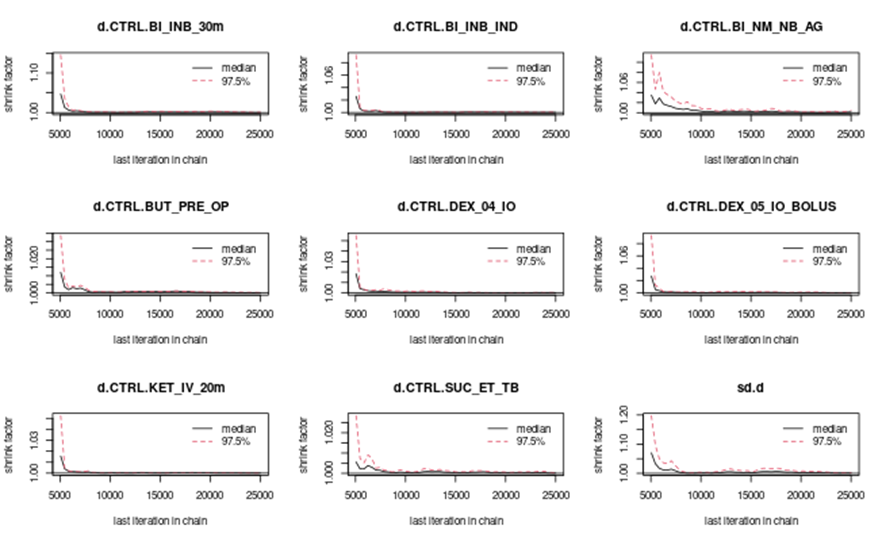


**Figure S15** Gelman convergence assessment plot for all studies in non-sevoflurane group.


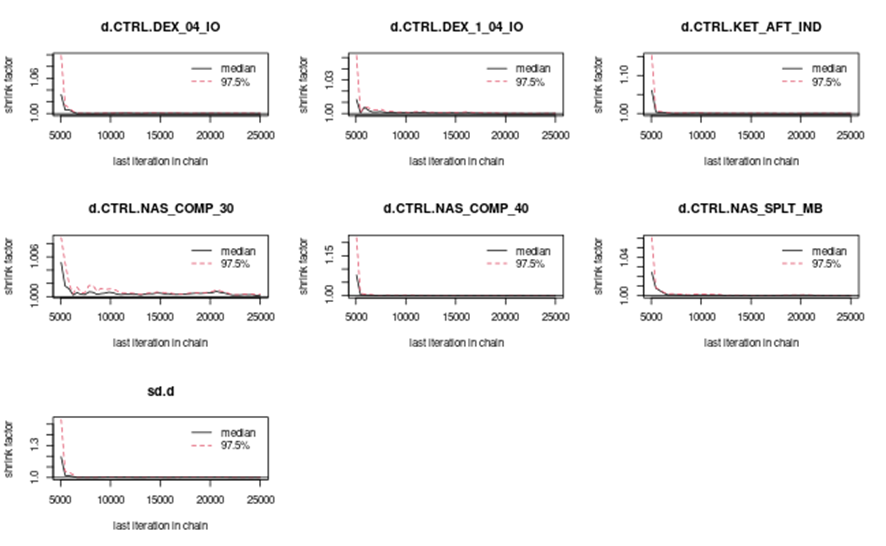

Supplement: Supplementary file 1 [file mmc1.docx]
